# A New Medical College in Atlanta

**Published:** 1878-12-20

**Authors:** 


### A NEW MEDICAL COLLEGE IN ATLANTA.

An application for a charter for a new Medical College in Atlanta has been made, and in a few days will be legally secured. We are authorized to state that prominent medical men in different sections of the South are moving in this matter, and that a Central Institution of a high order is contemplated.

A Board of Trustees have been appointed, consisting of the following gentlemen, to-wit:

| | |
|---|---|
| Hon. D W. Lewis, | Hon. Alex. H. Stephens, |
| Col. S. B. Hoyt, | Rev. W. F. Cook, |
| Col. G. T. Dodd, | A. J. Battle, LL. D., |
| Rev. C. W. Irwin, | Pres't Mercer Univ. |
| Mr. A. F. Hurt, | Thos. S. Powell, M. D. |
| Rev. David Butler, | R. C. Word, M, D. |
| W. T. Goldsmith, M. D., | Rev. H. C. Hornady, |
| Rev. J. J. Toon, | Mr. W. W. McAfee, |
| G. M. McDowell M. D. | |

